# An Unusual MRI Appearance of Osseous Metastases

**DOI:** 10.7759/cureus.300

**Published:** 2015-08-11

**Authors:** Konstantin Boroda, Ammar Chaudhry, Gary Clarke, Yudell Edelstein

**Affiliations:** 1 Radiology, Stony Brook University; 2 Internal Medicine, Albert Einstein College of Medicine; 3 Neuroradiology, Johns Hopkins University School of Medicine; 4 Pathology and Laboratory Medicine, Northport V.A. Medical Center

**Keywords:** bone metastases, bone metastases mri, cppd arthropathy, radiology bone metastases

## Abstract

Bone metastases can present in a wide variety of appearances across all imaging modalities. We present a unique appearance of a distal femoral metastasis in a patient who initially complained of knee pain. The radiographic and CT findings were initially suspicious for calcium pyrophosphate deposition (CPPD) arthropathy; however, an MRI demonstrated multiple lesions with a lamellated appearance confirmed on biopsy to be metastatic disease. This unusual lamellated appearance has not been previously described. We present this case to help distinguish this entity radiographically and better classify this finding as a manifestation of metastatic disease.

## Introduction

We present a patient with knee pain whose CT and radiography were suspicious for calcium pyrophosphate deposition CPPD arthropathy. However, further MR imaging revealed multiple bony lesions with a lamellated appearance, which has not been previously described in the literature for metastatic foci. Metastasis can present with a wide variety of findings depending on the primary neoplasm. However, the current finding of a lamellated appearance on MRI secondary to metastasis appears to be novel. We would like to emphasize this rare appearance of bone metastases and to distinguish it as a manifestation of metastatic disease.

## Case presentation

A 71-year-old male with a past medical history of hypertension, hyperlipidemia, diabetes mellitus, lumbar degenerative disc disease, and multiple cerebral vascular accidents presented with left knee pain. He believed that the knee pain first started after he syncopized from a hypoglycemic episode. He was hospitalized for the hypoglycemic episode and complained of knee pain during that admission. Knee radiographs were obtained but were interpreted as normal. Approximately one month later, the patient presented to his primary care provider with continuing symptoms of significant left knee pain. Physical examination of his knee revealed a joint effusion and tenderness to palpation. Informed patient consent was obtained prior to treatment.

### Imaging findings

**Figure 1:**
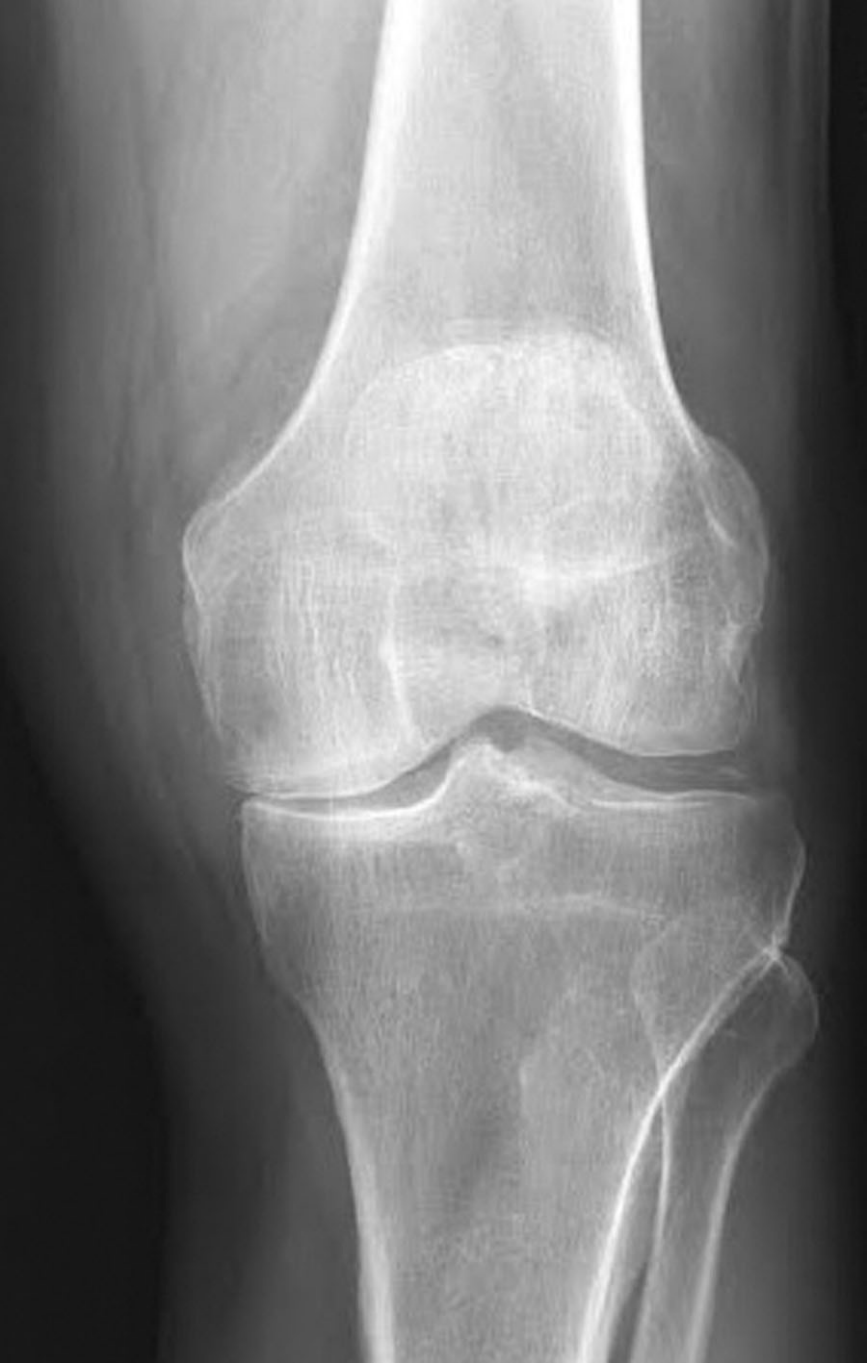
AP radiograph of the left knee

Radiography demonstrated joint space narrowing, subchondral sclerosis, subchondral lucency, and chondrocalcinosis, which raised suspicion for CPPD arthropathy (Figure 1).

**Figure 2:**
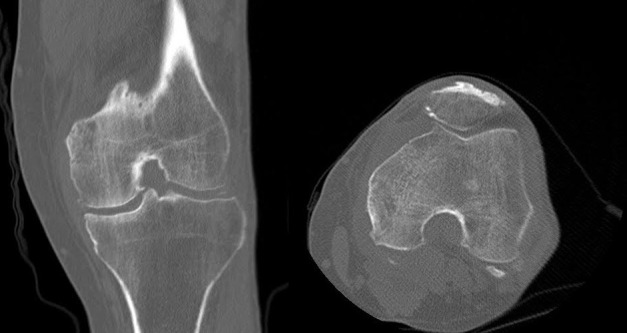
Coronal and axial CT images in bone window

A CT scan revealed a lesion in the medial femoral condyle with a faint target appearance of alternating lucency and sclerosis. There was also soft tissue fullness adjacent to the medial femoral condyle inseparable from the medial gastrocnemius muscle with linear calcification (Figure 2).

**Figure 3:**
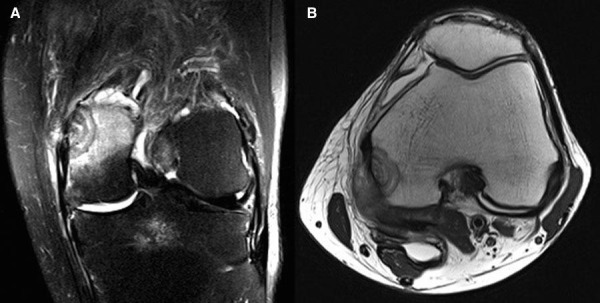
STIR sequence coronal image and T1 sequence axial image

MRI revealed two peripheral intramedullary focal mass-like lesions seen within the superior portion of the medial femoral condyle demonstrating a lamellated or “target” like appearance with extensive marrow and surrounding soft tissue edema (Figure 3A-3B).

**Figure 4:**
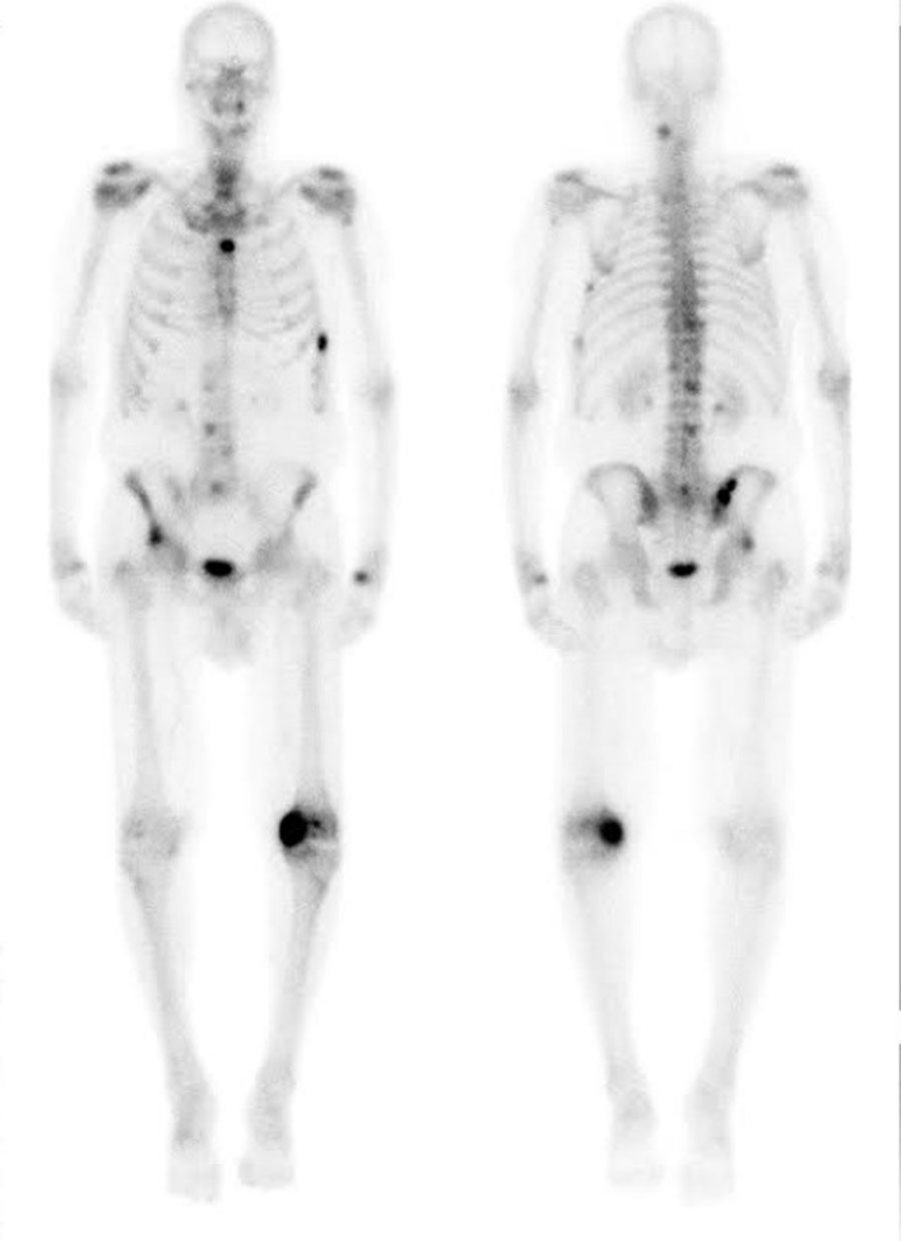
Technetium-99m whole body bone scan

A technetium-99mm bone scan demonstrated numerous foci of increased radiotracer uptake, including the left medial femoral condyle, left humerus, right iliac crest, right acetabulum, sternum, vertebrae, and ribs (Figure 4).

### Pathology, treatment, and outcome

**Figure 5:**
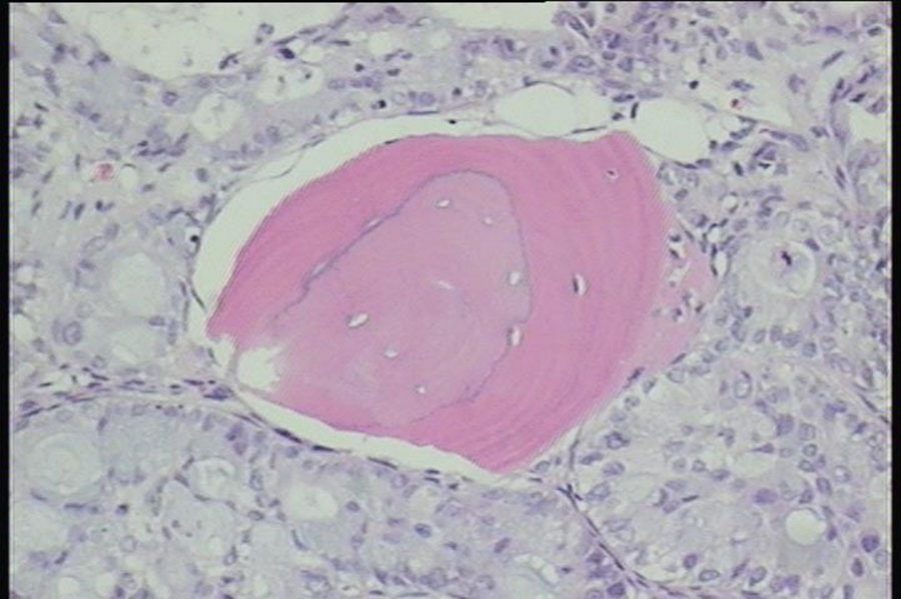
Hematoxylin and eosin stain at 100X

The decision was then made to perform an image-guided bone biopsy. Pathology revealed metastatic, moderately to well-differentiated adenocarcinoma. The immunoprofile supported metastatic origin from an upper gastrointestinal or pancreatobiliary tract primary tumor. The tumor marker, CA 19-9 was highly elevated to 289 U/mL (normal range, less than 37 U/L). CEA was also elevated to 223.85 ng/ml (normal range 0 - 5.0 ng/ml). The histological findings demonstrated a concentric pattern, which shared similarity with the lamellated appearance on MRI (Figure 5). The patient was initiated on chemotherapy but succumbed to his illness two months later.

## Discussion

Knee pain is a very common symptom encountered by primary care and emergency department providers. Most cases of knee pain are due to traumatic, inflammatory, and degenerative etiologies [1]. Knee radiography is usually the first step in the imaging evaluation of knee pain. However, advanced imaging is recommended when radiographic findings are suspicious for soft tissue injury or neoplastic disease [2].

The patient, in this case, had an intramedullary lesion within the medial femoral condyle. The most common differential diagnostic etiologies for multifocal intramedullary lesions included metastases, lymphoma, and multiple myeloma. In addition to metastatic bone lesions, patients with malignancy presenting with arthritic type pain may be suffering from paraneoplastic syndromes like hypertrophic osteoarthropathy, Sjogren’s syndrome, or carcinoma polyarthritis. Additionally, leukemia can cause hemarthrosis, gout, and synovial reaction.

Furthermore, gout can commonly occur in patients undergoing chemotherapy [3]. Both lymphoma and myeloma demonstrate low T1 signal and high signal on fluid-weighted sequences. Lymphoma can be lytic, sclerotic, or both. Multiple myeloma usually produces purely lytic lesions with or without endosteal scalloping, except in POEMS syndrome where sclerotic lesions are seen [4-5].

Metastatic disease is the most common neoplasm found in the bones. The skeleton (particularly the axial skeleton) is the third most common site for metastasis after the liver and lung. Approximately 80% of the primary tumors are adenocarcinomas arising from the prostate, breast, lung, kidney, thyroid, gastrointestinal tract, and bladder [6-7]. The most common primary tumor associated with metastatic carcinomatous arthritis is bronchogenic carcinoma [8-9]. Bronchial cancers are also the most common cause of distal metastases, beyond the elbow and knee [10]. The lesions found in this case were determined to be metastatic lung carcinoma. Lung carcinoma metastases usually appear lytic. Less often, lung lesions in bone may also be blastic [11-12].

We would like to highlight the unusual MRI appearance which demonstrated multiple concentric rings that can be described as a lamellated or target appearance. This was seen on both T2 fat-suppressed sequences and proton density sequences. These findings are not be confused with the Halo sign, which is a high-signal intensity ring surrounding a relatively low signal intensity on fluid-weighted (T2) images. It is thought to result from the destruction of trabeculae with a resultant fluid-filled gap, which is demonstrated as a retraction space on histology. A compliment to the Halo sign is the Bull’s Eye sign, which is a low signal intensity on T1 surrounding high-signal intensity. The Bull’s eye sign is thought to represent islands of developing bone marrow that contain yellow marrow centrally, which excludes malignancy [13].

Our histological images demonstrated concentric layers of necrotic bone, lamellar bone, and woven bone surrounded by tumor. The cause of the lamellated appearance on MRI may have been caused by waves of bone lysis followed by reparative “creeping” substitution. These histological findings are often seen in bone metastases. It is unknown why this MRI appearance is not encountered more often; but when it is seen, it should raise suspicion for metastatic disease.

## Conclusions

Metastatic disease is the most common neoplasm found in bones, but it can mimic a variety of entities on imaging. We present a unique MRI appearance of a bone metastasis. When encountered on MRI, this lamellated appearance should raise suspicion for metastatic disease.
